# Acute thrombosis in mitralic mechanical prosthesis: a case report

**DOI:** 10.1186/1757-1626-2-30

**Published:** 2009-01-08

**Authors:** Enrico Vizzardi, Antonio D'Aloia, Gregoriana Zanini, Elena Antonioli, Ermanna Chiari, Livio Dei Cas

**Affiliations:** 1 Section of Cardiovascular Disease, Department of Applied Experimental Medicine, Brescia University-Italy

## Abstract

We describe a case of a man, 42 years old, submitted to successful fibrinolitic strategy with rTPA in acute mitralic prosthesis valve thrombosis and ipomobility of one emidisk. There aren't a consensus agreement in therapeutic strategy but we may support the approach of some authors that employ fibrinolisis in patients without absolute or relative controindications and if thrombus dimension is less than 1 cm otherwise they make use of heparin therapy in non obstructive thrombosis with successive transesophageal echocardiography evaluation for the efficacy and the later indication for thrombolisis or surgery treatment.

## Introduction

The high incidence (7–34%/year) of thromboembolic risk in patients with valvular prosthesis without a correct administration of anticoagulant therapy has been demonstrated since the last 40 years. This risk is higher in patients older than 50 years old, with spherical prosthesis (actually not yet used), with prosthesis in mitralic location and in patients with association of atrial fibrillation [1-5]. However mechanical prosthesis have a long last and are more functional safely compared with the biological one; so mechanical devices are preferable implanted in young people even if they need a correct anticoagulation.

So some authors propose, as gold standard for oral anticoagulant therapy, to maintain INR (international normalized ratio) value between 2 and 3 (or 2.5–3.5) in all patients, between 3 and 4.5 in patients with old caged prosthesis and in association with antiplatlet therapy (ASA 100 mg) in patients with double prosthesis, ball devices, prosthetic valved conduit, patients with coronarosclerosis or by coronary artery bypass grafts in order to reduce thromboembolic risk [6].

## Case presentation

We describe a case of a man, 42 years old, that became to our emergency room for dyspnoea and tachycardia with fever (37.5°C) since two days with signs of congestive heart failure (pulmonary rantoli without ascites and pedal oedema).

The patient was carrier of a mitralic mechanical emidisc prosthesis (Sorin n°31 Bicarbon), implanted 7 years ago for mixomatosus valve with severe mitralic insufficiency from LA e LP mitral valve prolapse. The patient referred a lack in anticoagulant therapy with dicumarolic drug for about one week.

The electrocardiogram showed sinusal tachycardia (138 bpm) and left anterior bundle brunch emiblock.

Laboratory results demonstrated leukocytosis (17,5 × 1000/ul), inadequate values of scoagulation (INR 1.2, PT 13.1 sec, PT 98%), D-Dimer 569 ng/ml.

Chest radiograph described signs of pulmonary congestion. So the patients was treated with diuretics, inotropic agents at kidneys dosage and antibiotic e.v.

**Figure 1:**
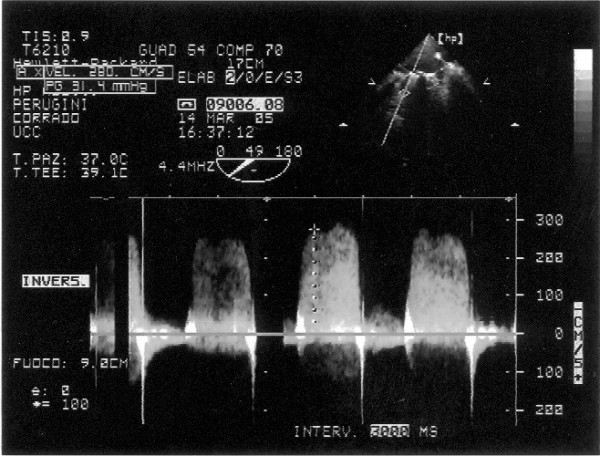
**Transesophageal echocardiography: Doppler CW on mitral prosthesis valve with the evidence of transvalvular gradient**.

**Figure 2:**
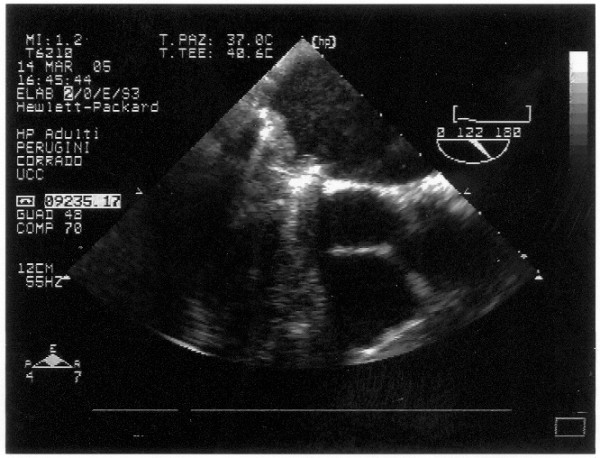
**Transesophageal echocardiography: 2D evidence of mitral prosthesis valve thrombosis**.

**Figure 3:**
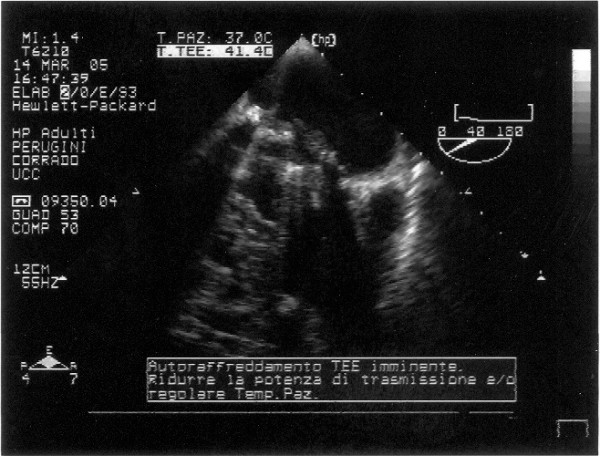
**Transesophageal echocardiography: 2D evidence of mitral prosthesis valve thrombosis**.

The transthoracic echocardiogram color Doppler showed a mechanical prosthesis in mitralic position with ipomobility of one emidisc and a transoesophageal one confirmed the presence of a thrombus on the atrial side, left atrial enlargement, trans prosthesis gradient major than 30 mmHg and medium 28 mmHg (fig. 1, 2, 3)

**Figure 4:**
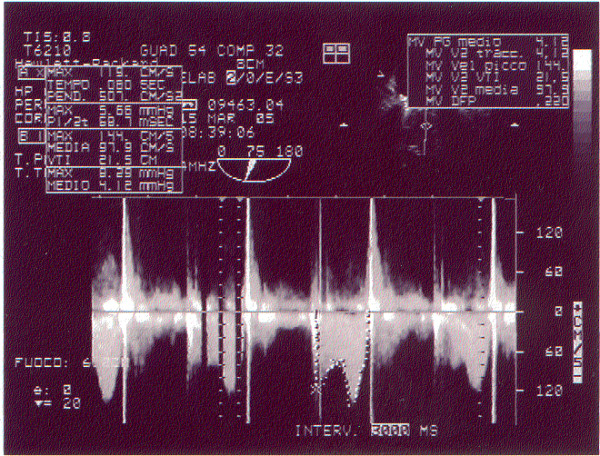
**Transesophageal echocardiography post fibrinolysis: CW on mitral prosthesis valve**. Significative reduction of transvalvular gradient.

In consideration of these reports the patient started a treatment with not fractionated heparin e.v. and fibrinolisis (rTPA) with improved in symptoms and hemodynamic signs. An echocardiogram color Doppler carried out after thrombolisis showed a marked reduction in transvalvular prosthetic gradient (ΔP medium 5 mmHg) confirmed by a transoesophageal echocardiography (fig. 4) that pointed out a resolution of the thrombotic image but evidenced the presence of two filaments moving towards the atrial side.

The patients started with oral anticoagulant therapy in association with the not fractioned heparin e.v until the achieved objective of an adequate anticoagulant value (INR between 2.5 and 3.5). after 10 days another echocardiogram color Doppler was performed with the evidence of complete resolution of prosthetic thrombosis.

## Discussion

Prosthetic thrombosis is a frightened complication of mechanical mitralic valve especially if there is a lack in the anticoagulant therapy.

The incidence of thrombosis in mechanical valve (and also in biological one) is between 0.1 and 5.7% pts/year in patients with uncorrected anticoagulant therapy [1-4].

The role of transesophageal echocardiography is essential to distinguish malfunctioning due to prosthesis obstruction or to prosthetic thrombosis with ipomobility of the mobile elements or thrombosis with preserved mobile elements excursion [7-10].

The prevalence of pannus formation in patients reoperated for prosthesis malfunction is about 25% and this anatomical evidence explain the vulnerability to thrombosis.

Medical and surgery strategy are the two possible options to treatment for acute thrombosis in prosthesis device [11-13].

Mortality percentage during surgery for prosthetic thrombosis is till 60% (in unstable hemodinamic patients and in emergency) while fibrinolitic agents involve a percentage of embolic complication between 9 and 15% with recurrent thrombosis in 16% of cases.

Active blooding, hemorrhagic stroke, recent skull trauma and uncontrolled high blood pressure are absolute controindications to fibrinolitic treatment; endocarditic processes, intracardiac large thrombus and recent surgery major operation or trauma are relative contraindication to thrombolisis [11-14].

Adequate treatment choice is related with clinical status (NYHA class), thrombus dimension, symptoms duration, prosthesis type (one disk versus bileaflets), elements ipomobility versus immobility and evidence of endocarditic process with abscess and thrombosis.

In our case we report the successful fibrinolitic strategy with rTPA in a patients with acute mitralic prosthesis valve thrombosis and ipomobility of one emidisk such as demonstrated in some study in patients with bidisk valve prosthesis: standard indications to thrombolisis in acute thrombosis are recent thrombus formation and only one disk blocked.

Surgery strategy is indicated in endocarditic process with abscess and large dimensions thrombus or in old date thrombus formation and if two disks prosthesis are involved.

In conclusion we could affirm that there aren't a consensus agreement in therapeutic strategy but we may support the approach of some authors that employ fibrinolisis in patients without absolute or relative controindications and if thrombus dimension is less than 1 cm otherwise they make use of heparin therapy in non obstructive thrombosis with successive transesophageal echocardiography evaluation for the efficacy and the later indication for thrombolisis or surgery treatment.

Surgery strategy should be reserved to patents with thrombus major than 1 cm, with or without endocarditis or in patients with controindications to thrombolisis.

## Consent

Written informed consent was obtained from the patient for publication of this case report and accompanying images. A copy of the written consent is available for review by the Editor-in-Chief of this journal.

## Competing interests

The authors declare that they have no competing interests.

## Authors' contributions

AD and EC performed transthoracic and thransesophageal echocardiography.

EV, GZ and EA treated and followed up the patient during the admission and the stay in the hospital. LDC gave us the supervision.
